# Tumor-Suppressive miR-192-5p Has Prognostic Value in Pancreatic Ductal Adenocarcinoma

**DOI:** 10.3390/cancers12061693

**Published:** 2020-06-25

**Authors:** Isabelle Flammang, Moritz Reese, Anda J. Strose, Zixuan Yang, Johannes A. Eble, Sameer A. Dhayat

**Affiliations:** 1 Department of General, Visceral and Transplantation Surgery, University Hospital Muenster, Albert-Schweitzer-Campus 1 (W1), 48149 Muenster, Germany; isabelle.flammang@ukmuenster.de (I.F.); m_rees04@uni-muenster.de (M.R.); anda.stroese@web.de (A.J.S.); yzxbcchina@gmail.com (Z.Y.); 2 Department of Physiological Chemistry and Pathobiochemistry, University of Muenster, Waldeyerstrasse 15, 48149 Muenster, Germany; johannes.eble@uni-muenster.de

**Keywords:** pancreatic ductal adenocarcinoma, microRNA-192-5p, epithelial-to-mesenchymal transition, liquid biopsy, exosomes, zinc finger E-box-binding homeobox 2

## Abstract

Pancreatic ductal adenocarcinoma (PDAC) is characterized by fast tumor progression and diagnosis at advanced, inoperable stages. Previous studies could demonstrate an involvement of miR-192-5p in epigenetic regulation of visceral carcinomas. Due to contradictory results, however, the clinical utility of miR-192-5p in PDAC has yet to be determined. MiR-192-5p expression was analyzed by RT-qRT-PCR in human PDAC and benign tissue (*n* = 78), blood serum (*n* = 81) and serum exosomes (*n* = 74), as well as in PDAC cell lines (*n* = 5), chemoresistant cell clones (*n* = 2), and pancreatic duct cell line H6c7. Analysis of EMT-associated (epithelial-to-mesenchymal transition) proteins was performed by immunohistochemistry and Western blot. MiR-192-5p was deregulated in PDAC as compared to healthy controls (HCs), with downregulation in macrodissected tissue (*p* < 0.001) and upregulation in blood serum of PDAC UICC (Union for International Cancer Control) stage IV (*p* = 0.016) and serum exosomes of PDAC UICC stages II to IV (*p* < 0.001). MiR-192-5p expression in tumor tissue was significantly lower as compared to corresponding peritumoral tissue (PDAC UICC stage II: *p* < 0.001; PDAC UICC stage III: *p* = 0.024), while EMT markers ZEB1 and ZEB2 were more frequently expressed in tumor tissue as compared to peritumoral tissue, HCs, and chronic pancreatitis. Tissue-derived (AUC of 0.86; *p* < 0.0001) and exosomal (AUC of 0.83; *p* = 0.0004) miR-192-5p could differentiate between PDAC and HCs with good accuracy. Furthermore, high expression of miR-192-5p in PDAC tissue of curatively resected PDAC patients correlated with prolonged overall and recurrence-free survival in multivariate analysis. In vitro, miR-192-5p was downregulated in gemcitabine-resistant cell clones of AsPC-1 (*p* = 0.029). Transient transfection of MIA PaCa-2 cells with miR-192-5p mimic resulted in downregulation of ZEB2. MiR-192-5p seems to possess a tumor-suppressive role and high potential as a diagnostic and prognostic marker in PDAC.

## 1. Introduction

Pancreatic ductal adenocarcinoma (PDAC) remains to be one of the most lethal malignant diseases in western countries. The five-year relative survival rate of PDAC is less than 10% and this is mainly attributed to its high invasiveness and fast tumor progression [1,2]. As a result of innovative diagnostic tools, pharmaceuticals, and surgical techniques, considerable advances have been achieved in the management of multiples types of cancer. However, no substantial improvements have yet been registered for PDAC. Therapy of PDAC is interdisciplinary and multimodal, while surgical resection currently represents the only treatment option potentially enabling long-term survival. Due to a lack of disease-specific symptoms, however, diagnosis of early-stage PDAC is often incidental and patients usually present with advanced, inoperable disease, leaving no possibility for curation. Gemcitabine and FOLFIRINOX (folinic acid, fluorouracil, irinotecan, oxaliplatin) are commonly administered chemotherapeutic regimens in the adjuvant and palliative management of PDAC and have been shown to slightly improve the prognostic outcome of patients. Nevertheless, response rates to chemotherapy are reported to be as low as 9.4–31.6% in the metastatic setting, yet another reason for the fatal prognosis of the disease [3,4,5].

To date, carbohydrate antigen 19-9 (CA.19-9) is the preferred blood-derived tumor marker of PDAC, however, there are several limitations towards its clinical utility as a biomarker for early detection or screening of PDAC. CA.19-9 often fails to reliably differentiate between chronic pancreatitis (CP) and PDAC and is frequently elevated in patients with obstructive jaundice as a result of benign gastrointestinal diseases such as liver cirrhosis or choledocholithiasis. Moreover, expression of CA.19-9 is conditional upon a Lewis-positive blood phenotype, resulting in false-negative diagnostic results among the 5–10% of the population that lack expression of Lewis antigens [6]. In light of these circumstances and the lack of successful therapeutic innovations, one of the most promising strategies in the fight against PDAC seems to be the identification of biomarkers (1) that allow for accurate diagnosis of PDAC at early, resectable stages and (2) that can be reliably correlated with treatment response and prognosis of patients.

Over the last two decades, microRNAs (miRs) have increasingly raised the attention of researchers as regulators of gene expression at the post-transcriptional level with impact on key signaling pathways in tumorigenesis and progression of almost every type of cancer including PDAC [7,8]. They are small, non-coding, highly conserved RNA (ribonucleic acid) molecules with a length of approximately 22 nucleotides and—depending on their target genes—can possess either oncogenic or tumor suppressive functions [9,10]. This has led to the development of a whole new class of anticancer drugs—miR therapeutics [11,12]. Cancer tissue-derived miRs in particular have been attributed regulatory functions in the formation and progression of cancer. In PDAC, we have recently reported on the tumor suppressive role of the miR-200-family (miRs 141, 200a, 200b, 200c, and 429) as inhibitors of EMT, a vital process preceding metastatic dissemination [13,14]. In contrast, miRs 21, 23a, and 27a for example were found to hold oncogenic properties, inhibiting multiple tumor suppressor genes, hence promoting tumor progression [15]. In addition, we could previously reveal an involvement of several miRs including aforementioned miR-21 in gemcitabine-resistance in PDAC [16].

Next to their regulatory functions, the deregulation of specific miRs across various types of clinical specimens has also made them attractive biomarker candidates in cancer research. Specifically in PDAC, serum-derived circulating miRs were found to be of high diagnostic and prognostic value, often in combination with other biomarkers or as part of a miR panel [17,18]. However, miRs can also enter circulation encapsulated within exosomes.

Exosomes are nano-sized membrane vesicles of endocytic origin with a diameter of about 30–100 nm that contribute to intercellular signaling and communication [19]. They are secreted by almost all types of cells including pancreatic tumor cells and they contain a specific cargo of proteins, messenger RNAs and miRs, providing a rich potential source of biomarkers [20]. Similarly to circulating miRs, exosomal miRs were found to be deregulated and of diagnostic and prognostic value in PDAC. MiRs let7a, 122, and 221-3p were found to be downregulated, while miRs 10b, 17-5p, 19a-3p, 19b-3p, 20a, 21, 30c, 106b, 122-5p, 181a, 191, 192-5p, 193-3p, 196a, 200b, 200c, 451a, 1246, 3976, 4306, 4644 were found to be overexpressed in plasma- or serum-exosomes of PDAC patients [21,22,23,24,25,26,27,28,29].

Recent studies suggest that miR-192-5p in particular is involved in PDAC progression with aberrant expression in tumor tissue, blood serum, and serum-derived exosomes of PDAC patients. However, results concerning the directionality of its deregulation were contradictory [28,30,31]. In this study, we explored the expression of miR-192-5p in human PDAC cell lines in vitro as well as regulation of well-known EMT markers by transient transfection with miR-192-5p. Additionally, we analyzed the diagnostic and prognostic value of miR-192-5p in PDAC tissue, blood serum, and serum exosomes of patients with PDAC UICC (Union for International Cancer Control) stages II–IV, chronic pancreatitis, pancreatic cystadenoma, and healthy controls (HCs).

## 2. Results

### 2.1. Expression of miR-192-5p and EMT-Related Proteins in Tumor Cells of PDAC

**Figure 1:**
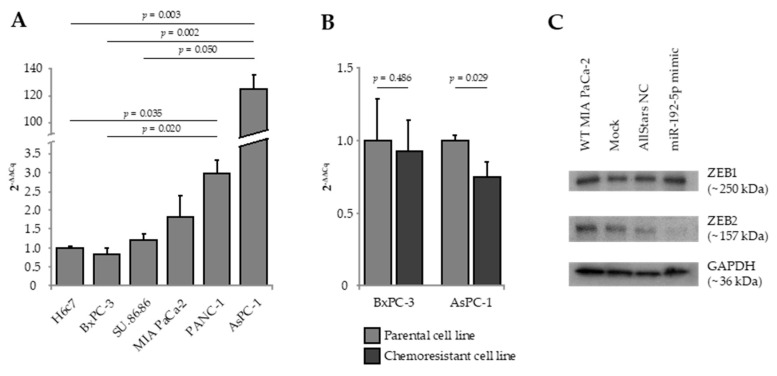
(**A**) Expression of miR-192-5p in five human pancreatic ductal adenocarcinoma (PDAC) cell lines relative to the human pancreatic duct epithelial cell line H6c7. (**B**) Expression of miR-192-5p in gemcitabine-resistant cell clones relative to parental gemcitabine-sensitive cells of BxPC-3 and AsPC-1. Data were plotted as 2^−ΔΔCq^ ± standard error of the mean (SEM). Statistical significance was assumed at *p* ≤ 0.05 (**A**) Kruskal–Wallis test, (**B**) Mann-Whitney *U* test. (**C**) Expression of epithelial-to-mesenchymal transition-associated marker proteins ZEB1 and ZEB2 in MIA PaCa-2 cells following mock transfection and transfection with Allstars negative control and miR-192-5p mimic. Detailed information about western blot can be found at Figures S2 and S3.

Expression of miR-192-5p, E-cadherin, Vimentin, ZEB1, and ZEB2 were quantified by real-time quantitative reverse transcription polymerase chain reaction (RT-qRT-PCR) in five established human PDAC cell lines as well as in the human pancreatic duct epithelial cell line H6c7 (Figure 1A). AsPC-1, metastasized PDAC cells derived from malignant ascites, presented with a significant overexpression of miR-192-5p as compared to BxPC-3 cells (solid primary PDAC; 2^-ΔΔCq^ = 145.16; *p* = 0.002), SU.86.86 cells (solid liver metastasis of PDAC; 2^-ΔΔCq^ = 102.44; *p* = 0.050), and H6c7 cells (2^-ΔΔCq^ = 125.03; *p* = 0.003). As compared to H6c7 cells, expression of epithelial marker E-cadherin was significantly lower in MIA PaCa-2 and PANC-1 cells, while mesenchymal marker Vimentin was significantly overexpressed in MIA PaCa-2, PANC-1, and AsPC-1 cells. Relative to H6c7 cells, ZEB1 was overexpressed in AsPC-1 cells and ZEB2 was overexpressed in PANC-1 and BxPC-3 cells (Figure S1).

Next, we quantified the expression of miR-192-5p in in vitro-generated gemcitabine-resistant cell clones of parental AsPC-1 and BxPC-3 cells. Interestingly, gemcitabine resistance of AsPC-1 cells was accompanied by significant downregulation of miR-192-5p (2^−ΔΔCq^ = 0.74; *p* = 0.029; Figure 1B). The difference in miR-192-5p expression between gemcitabine-sensitive and gemcitabine-resistant clones of BxPC-3 was statistically not significant.

### 2.2. Transient Transfection of PDAC Cells with miR-192-5p Mimic

To elucidate a potential role of miR-192-5p as a regulator of EMT in PDAC, we overexpressed miR-192-5p in MIA PaCa-2 cells by transient transfection. Next, we analyzed the effect of transfection on expression of EMT-associated transcription factors ZEB1 and ZEB2 (zinc finger E-box-binding homeobox 1/2) by Western blot. While there was no effect on expression of ZEB1, expression of ZEB2 in MIA PaCa-2 cells transfected with miR-192-5p mimic was clearly decreased, indicating that ZEB2 seems to be a direct target of miR-192-5p (Figure 1C).

### 2.3. Clinicopathological Patient Data

In total, 168 patients were included in this study, thereof 81 patients with PDAC, 38 patients with CP, 11 patients with intraductal papillary mucinous neoplasm (IPMN), 19 patients with benign cystadenoma, and 19 aged-matched volunteers as HCs. The 81 PDAC patients were further divided into subgroups according to the 8th edition of the UICC TNM (Tumor, Nodes, Metastasis) classification of malignant tumors. Major characteristics of all 168 patients are summarized in Table 1. These were homogenously distributed between the investigated study groups except for gender (*p* = 0.034), pre-surgical chronic pancreatitis (*p* < 0.001), and pre-surgical levels of circulating CA.19-9 (*p* < 0.001), CEA (carcinoembryonic antigen, *p* < 0.013), and bilirubin (*p* = 0.001).

Clinicopathological and follow-up data of 42 PDAC patients treated with curative intent are shown in Table 2. Univariate survival analysis for various clinicopathological factors connected to PDAC pathology revealed a correlation between low tumor grading (*p* = 0.024) as well as administration of adjuvant chemotherapy (*p* = 0.013) and prolonged PDAC-specific overall survival (OS) of curatively resected patients. Administration of adjuvant chemotherapy was also associated with improved recurrence-free survival (RfS) (*p* = 0.025). Though not significant, there seemed to be a tendency towards an association of shorter RfS with high tumor grading (*p* = 0.065), lymphatic invasion (*p* = 0.056), and positive resection margin (*p* = 0.052) as well.

### 2.4. Expression of miR-192-5p in Tumoral and Peritumoral Tissue

**Figure 2:**
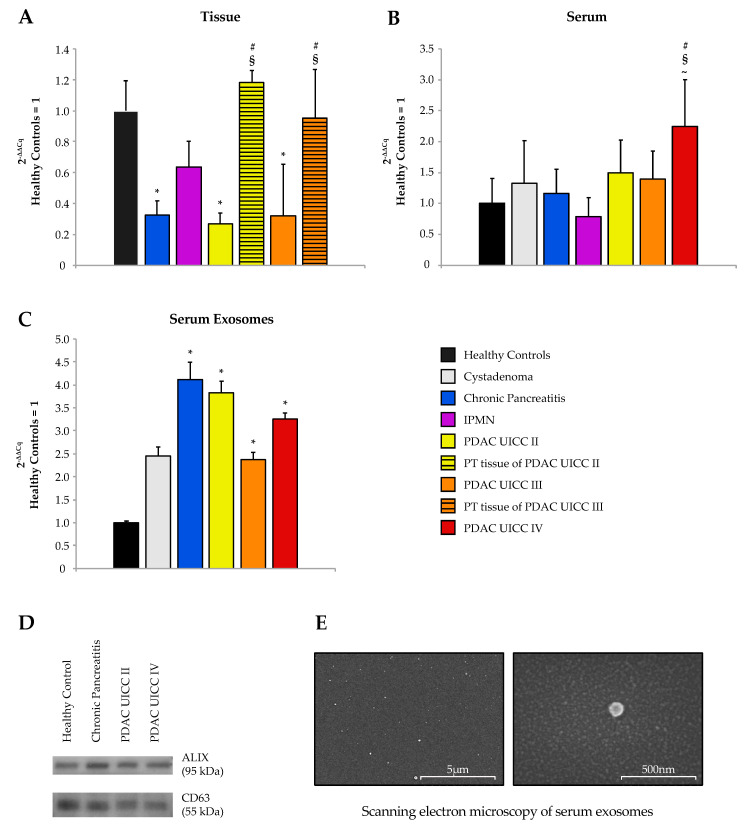
Expression of miR-192-5p in (**A**) tissue, (**B**) serum, and (**C**) serum exosomes. Data are plotted as 2^−ΔΔCq^ ± standard error of the mean (SEM) and relative to healthy controls. Statistical significance (assumed at *p* ≤ 0.05, Kruskal–Wallis test) is indicated as follows: to healthy controls (*), to chronic pancreatitis (#), to intraductal papillary mucinous neoplasm (IPMN) (~), and to tumoral tissue of the same UICC (Union for International Cancer Control) stage (§). (**D**) Western blot for exosomal markers ALIX (apoptosis-linked gene 2—interacting protein X) and CD63 (cluster of differentiation 63) on exosome samples purified from patients’ blood serum. (**E**) Exemplary images of scanning electron microscopy of CD63 positive serum exosomes at magnifications of 10,000 and 100,000. Detailed information about western blot can be found at Figures S2 and S3.

Expression of miR-192-5p was analyzed by RT-qRT-PCR in formalin-fixed paraffin-embedded (FFPE) tissue of 78 patients, thereof 22 patients with CP, nine patients with IPMN, 24 patients with PDAC UICC stage II, and seven patients with PDAC UICC stage III. Surgically removed PDAC tissue specimens were macrodissected to obtain tumoral as well as peritumoral (PT) tissue punches. Matching specimens of tumoral and adjacent PT tissue were sampled for 26 PDAC patients. Tissue of 16 patients with benign serous or mucous cystadenoma were included as a control group with tissue punches taken in clear distance from the benign adenoma. MiR-192-5p was significantly downregulated in PDAC tumors as compared to healthy tissue (UICC II: *p* < 0.001; *r* = 0.587; UICC III: *p* = 0.01; *r* = 0.539) and adjacent PT tissue (UICC II: *p* < 0.001; *r* = 0.661; UICC III: *p* = 0.024; *r* = 0.585; Figure 2A).

### 2.5. Expression of Epithelial and Mesenchymal Markers in Pancreatic Tissue

**Figure 3:**
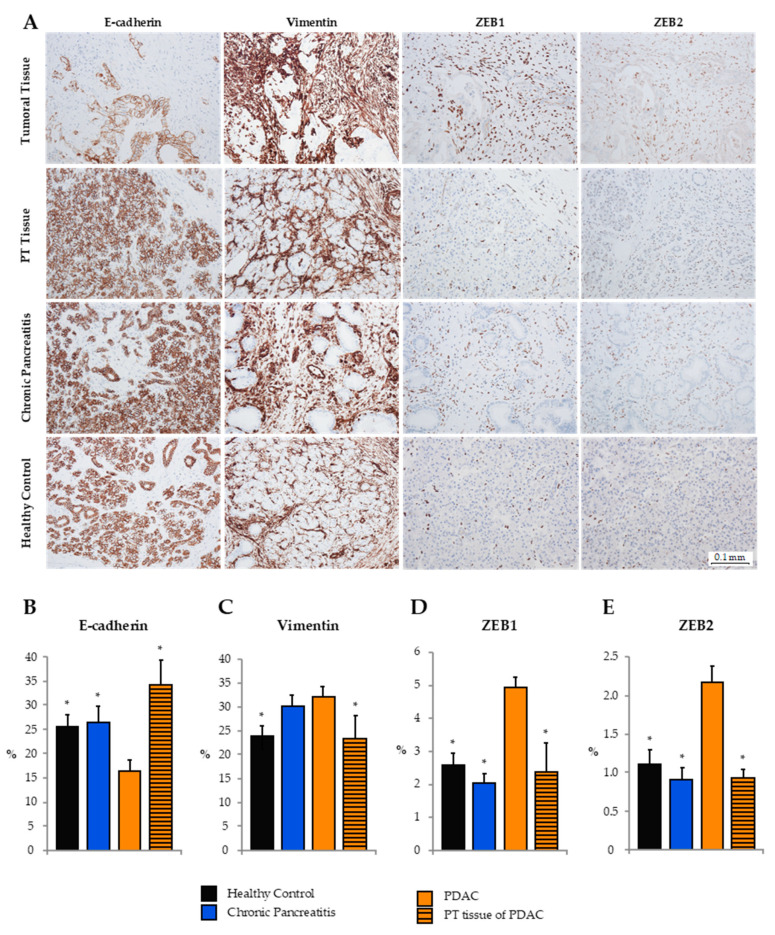
(**A**) Tissue microarray immunohistochemistry of exemplary PDAC, chronic pancreatitis (CP), and healthy clinical tissue samples. Mean expression ± standard error of the mean (SEM) of epithelial-to-mesenchymal transition markers (**B**) E-cadherin, (**C**) Vimentin, (**D**) ZEB1, and (**E**) ZEB2 as assessed by ImageJ. (*) indicates statistically significant differences in expression to PDAC tumor tissue (assumed at *p* ≤ 0.05, Kruskal–Wallis test). PT, peritumoral.

As shown previously, analysis of clinical FFPE tissue samples of human PDAC by RT-qRT-PCR revealed an upregulation of miR-192-5p in PT versus tumoral tissue of PDAC UICC stages II and III. Next, we evaluated the expression of epithelial (E-cadherin) and mesenchymal (Vimentin, ZEB1, ZEB2) markers by tissue microarray immunohistochemistry in tumoral (*n* = 28) and PT (*n* = 10) tissue, as well as tissue of CP (*n* = 16) and HCs (*n* = 10; Figure 3). Expression of E-cadherin in tumoral PDAC tissue was significantly lower than in PT tissue and tissue of CP and HCs. In contrast, Vimentin was mainly expressed in stromal cells of PDAC and CP, while expression in healthy and peritumoral tissue was relatively low. ZEB1 and ZEB2, known to be transcriptional blockers of E-cadherin, were almost exclusively expressed in the nuclei of stromal cells. Hence, ZEB1 and ZEB2 were more frequently expressed in tumor tissue as compared to PT tissue or tissue of CP and HCs.

### 2.6. Expression of miR-192-5p in Blood Serum and Serum Exosomes

Expression of circulating miR-192-5p was analyzed by RT-qRT-PCR in serum samples of 81 study participants, consisting of 14 HCs, six patients with benign cystadenoma, 13 patients with CP, six patients with IPMN, and 42 patients with PDAC (13× UICC II, 15× UICC III, 14 ×UICC IV; Figure 2B). Significant differences in levels of circulating miR-192-5p were detected for PDAC UICC stage IV as compared to HCs (2^−ΔΔCq^ = 2.24, *p* = 0.016, *r* = 0.45), CP (2^−ΔΔCq^ = 1.92, *p* = 0.033, *r* = 0.411), and IPMN (2^−ΔΔCq^ = 2.85, *p* = 0.006, *r* = 0.610). There were no further significant differences for any pairwise comparison of before mentioned study groups.

The study cohort for total serum exosomes included 12 HCs, seven patients with benign cystadenoma, 11 patients with CP, and 44 patients with PDAC (15 x UICC II, 16 x UICC III, 13 x UICC IV; Figure 2C). Statistically significant differences were apparent comparing HCs with CP (2^−ΔΔCq^ = 4.11, *p* = 0.003, *r* = 0.613), PDAC UICC stage II (2^−ΔΔCq^ = 3.83, *p* = 0.001, *r* = 0.663), PDAC UICC stage III (2^−ΔΔCq^ = 2.38, *p* = 0.024, *r* = 0.428), and PDAC UICC stage IV (2^−ΔΔCq^ = 3.25, *p* = 0.002, *r* = 0.631). Furthermore, significant differences were detected when comparing non-metastasized PDAC (UICC II+ III: 2^−ΔΔCq^ = 2.99, *p* = 0.001, *r* = 0.49) and all 44 PDAC samples (UICC II, III, IV: 2^−ΔΔCq^=3.07, *p* < 0.001, *r* = 0.469) with HCs. There were no significant differences in exosomal expression of miR-192-5p between metastasized and non-metastasized PDAC samples. Exosomal expression of miR-192-5p was higher in patients with benign cystadenoma as compared to HCs, albeit not significant (*p* = 0.062). The presence of exosomes in exosome-enriched serum samples was verified by Western blot analysis using exosomal markers CD63 (cluster of differentiation 63) and ALIX (apoptosis-linked gene 2—interacting protein X, Figure 2D) and scanning electron microscopy (Figure 2E).

### 2.7. Diagnostic Analysis of Tissue and Circulating miR-192-5p

**Figure 4:**
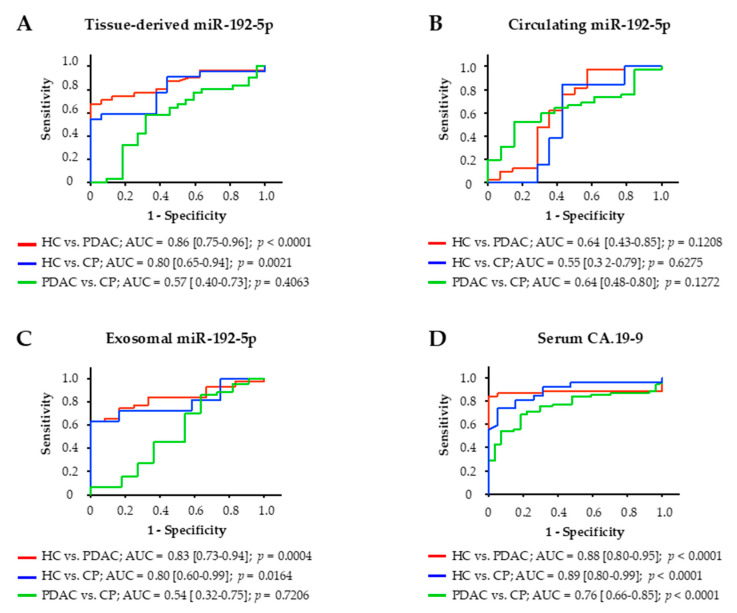
Receiver operating characteristic (ROC) curve analysis for diagnostic potential of miR-192-5p in (**A**) tissue, (**B**) serum, (**C**) serum exosomes, and for (**D**) carbohydrate antigen 19.9. AUC, area under the ROC curve; CP, chronic pancreatitis; HC, healthy controls; PDAC, pancreatic ductal adenocarcinoma.

The diagnostic potential of miR-192-5p derived from tissue, serum, and serum exosomes of study participants was assessed by construction of receiver operating characteristic (ROC) curves and calculation of the area under the ROC curve (AUC; Figure 4). Among these three clinical specimens, expression of miR-192-5p in tissue was most accurate in distinguishing PDAC patients from HCs with an AUC of 0.86 (*p* < 0.0001). Tissue-derived miR-192-5p could also differentiate between HCs and patients with CP (AUC of 0.80; *p* = 0.0021) but not between PDAC patients and patients with CP (AUC of 0.57; *p* = 0.4063). Similar results could be achieved for miR-192-5p derived from serum exosomes. Exosomal miR-192-5p could distinguish HCs from PDAC patients (AUC of 0.83, *p* = 0.0004) and from patients with CP (AUC of 0.80; *p* = 0.0164) but could not differentiate between PDAC patients and patients with CP either (AUC of 0.54; *p* = 0.7206). Circulating miR-192-5p could not differentiate between any of these three groups of patients. Tumor marker CA.19-9 slightly outperformed exosomal and tissue-derived miR-192-5p in distinguishing HCs from PDAC patients (AUC of 0.88; *p* < 0.0001) as well as from patients with CP (AUC of 0.76; *p* < 0.0001) but in contrast to miR-192-5p was also able to differentiate between PDAC patients and patients with CP (AUC of 0.76; *p* < 0.0001).

### 2.8. Prognostic Value of Tissue and Circulating miR-192-5p

**Figure 5:**
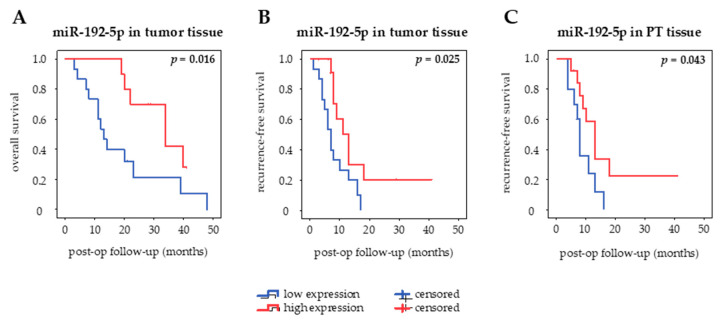
Kaplan–Meier curves. Prognostic impact of tissue-derived miR-192-5p on (**A**) overall survival and (**B**) recurrence-free survival and of (**C**) peritumoral tissue-derived miR-192-5p on recurrence-free survival in curatively resected patients. Bold values indicate statistical significance (*p* ≤ 0.05, log-rank test).

The prognostic value of miR-192-5p in different clinical specimens of curatively resected PDAC patients was assessed using Kaplan–Meier curves and log-rank test (Figure 5). MiR-192-5p was categorized into high and low expression based on a cut-off defined as the median value of expression in each type of sample. There was no association of miR-192-5p expression with OS or RfS in serum and serum exosomes. However, there seemed to be a tendency towards an inverse correlation of circulating serum levels of miR-192-5p and OS (*p* = 0.383) as well as RfS (*p* = 0.236). High expression of miR-192-5p in PT tissue was associated with prolonged RfS (*p* = 0.043, median RfS 13 months (high) vs. 8 months (low)) but not OS (*p* = 0.537), whereas high expression of miR-192-5p in tumor tissue correlated both with longer OS (*p* = 0.016, median OS 34 months (high) vs. 13 months (low)) and RfS (*p* = 0.025, median RfS 13 months (high) vs. 7 months (low)).

In order to adjust for potential confounders, a Cox proportional-hazards model was applied to assess the independent prognostic value of miR-192-5p. Univariate survival analysis revealed no correlation of parameters age, BMI, smoking, alcohol abuse, pre-surgical diabetes and pancreatitis, CA.19-9, CEA, and bilirubin with OS and RfS. Among the dichotomized histopathologic variables, high tumor grading was associated with shorter OS (*p* = 0.038) (Table 3). Moreover, administration of adjuvant chemotherapy had a significant benefit on OS (*p* = 0.021) and RfS (*p* = 0.038). As for expression of miR-192-5p in the four different clinical specimens, tumor tissue was the only subgroup, in which miR-192-5p expression could be correlated with the prognosis of patients (OS: *p* = 0.024; RfS: *p* = 0.038). Significant variables from the univariate analysis were further investigated in the multivariate analysis. In regard to OS, low tumor grading (*p* = 0.008) and high expression of miR-192-5p in tumor tissue (*p* = 0.006) were revealed as being potentially independent prognostic factors. As for RfS, high expression of miR-192-5p in tumor tissue (*p* = 0.045) and adjuvant administration of chemotherapy (*p* = 0.050) could be identified as potentially independent favorable prognostic biomarkers.

## 3. Discussion

In this study, downregulation of miR-192-5p in PDAC tissue shows great potential as an independent prognostic marker for curatively resected patients as revealed by multivariate survival analysis. Moreover, tissue-derived and serum-exosomal expression of miR-192-5p could discriminate between PDAC and HCs with good accuracy, similar to that of established tumor marker CA.19-9. However, the clinical value of miR-192-5p remains ambiguous, as previous studies investigating its regulation in PDAC revealed in part contradictory results (Table S1 [27,28,30,31,32,33,34,35,36,37,38,39,40,41,42,43,44,45,46,47,48]). For the first time in 2013, Zhao et al. described an overexpression of miR-192-5p in tumor tissue and blood serum of PDAC patients [30]. These data were supported by comparable results of Zou et al. in 2019, who additionally revealed an overexpression of miR-192-5p in serum exosomes and both studies concluded that serum-derived miR-192-5p alone has little relevance as a diagnostic or prognostic marker [28]. Even so, Zhao et al. later reported in a follow-up study that circulating miR-192-5p in combination with miR-194 significantly increased its ability to differentiate between PDAC and HCs (AUC = 0.807 vs. 0.627) [49]. Additionally, a study by Zhou et al. concluded that miR-192-5p as part of a six-miR-panel of upregulated plasma miRs reached an excellent accuracy in discriminating PDAC from HCs (AUC = 0.934) [27].

By contrast, Botla et al. demonstrated a downregulation of miR-192-5p in tumor tissue with excellent discrimination between PDAC and HCs and decreased overall survival, emphasizing the tumor suppressive function of miR-192-5p in PDAC [31]. Our present study results confirm this role of miR-192-5p as a tumor suppressor in PDAC. Additionally, we could observe a downregulation of tissue-derived miR-192-5p and an upregulation of exosomal miR-192-5p in patients with CP, reflecting the multifactorial involvement of deregulated miRs not only in malignant but also inflammatory pathologies of the pancreas. In this study, serum-derived expression of miR-192-5p was significantly upregulated in PDAC patients with metastasized disease. As compared to HCs, however, the difference was not statistically significant in patients with PDAC UICC II and III. In regard to a possible correlation between expression of circulating miR-192-5p and metachronous distant metastasis, further subgroup analyses of non-metastasized early tumor stages are required.

Apart from PDAC, expression of miR-192-5p was also reported to be downregulated in various other tumor entities, such as breast cancer, osteosarcoma, and bladder cancer [32,33,34,35]. Conversely, expression of miR-192-5p is upregulated in tumor tissue and plasma of patients with periampullary carcinomas and correlates with poor survival of UICC stage III patients [36]. Furthermore, decreased levels of circulating miR-192-5p were shown to correlate with presence of bone metastasis in non-small cell lung cancer (NSCLC) [37]. Contrarily, its upregulation in tissue of esophageal squamous cell carcinoma, prostate cancer, and nasopharyngeal carcinoma illustrated the different tumor specific signatures of miR regulation [38,39,40].

In vitro, miR-192-5p activates cell apoptosis and inhibits tumor cell proliferation, migration, and invasion in multiple tumor entities such as breast cancer, colon cancer, lung cancer, osteosarcoma, bladder cancer, melanoma, and PDAC [31,32,33,35,37,41,42,43,44]. Additionally, overexpression of miR-192-5p in cell lines of breast cancer and gastric cancer increased the sensitivity to chemotherapeutic agents doxorubicin and cisplatin, respectively [45,46], corresponding to our results showing a downregulation of miR-192-5p in gemcitabine-resistant PDAC cell lines. Furthermore, in vitro analyses in the present study revealed an extraordinary upregulation of miR-192-5p in a PDAC cell line derived from ascites metastasis in contrast to other epithelial cell lines of solid primary and metastatic PDAC and H6c7, a benign epithelial cell line derived from normal human pancreatic duct epithelium (HPDE). Regarding this supposed discrepancy with upregulation of miR-192-5p in malignant ascites, blood serum, and circulating exosomes on the one hand and downregulation in tumor tissue and solid tumor cell lines on the other hand, we previously discussed the miR-200 family as a tumor suppressive regulator of EMT in PDAC [13,29]. In accordance with immunohistological analyses of Botla et al., we could demonstrate that miR-192-5p is involved in the process of EMT as well. Transfectional upregulation of miR-192-5p resulted in the suppression of ZEB2, which is known to induce EMT by repressing genes of various epithelial cell–cell junctions such as E-cadherin [50,51]. No significant reciprocal effect could be observed by inhibition of miR-192-5p in MIA PaCa-2 cells. We suggest that this could in part be attributed to the low intrinsic expression of miR-192-5p in MIA PaCa-2 cells. However, further preclinical studies are required to fully understand the role of miR-192-5p, its targets and involvement in the complex gene regulatory network of EMT.

Taken together, tumor suppressive miR-192-5p seems to be involved in EMT-regulation with high potential as a diagnostic and prognostic marker in PDAC. In contrast to liquid biopsies, expression of tissue-derived miR-192-5p with prognostic value would only be available after surgical resection. Even so, we believe that quantification of miR expression from tumor and PT tissue could enhance risk stratification of PDAC patients. This in turn could facilitate the selection of suitable adjuvant treatment regimens and adjustment of time intervals for follow-up and aftercare, leading to a more personalized management of PDAC. Therefore, multicenter and predictive studies are required to define the suitability of miR-192-5p as a clinical biomarker and therapeutic target.

## 4. Materials and Methods

### 4.1. Cell Culture and Transient Transfection

Human pancreatic duct epithelial cell line H6c7 and human PDAC cell lines AsPC-1, BxPC-3, MIA PaCa-2, PANC-1, and SU.86.86 were obtained from the American Type Culture Collection (ATCC; Manassas, VA, USA). All cells were cultured and subcultured in accordance with the ATCC’s instructions. Chemoresistant cell clones were established by continuous treatment of parental cell lines while gradually increasing concentrations of gemcitabine (Gemzar^®^; Eli Lilly, Indianapolis, IN, USA), starting with the individual IC50 of parental cells as previously reported [16]. Transient transfection of MIA PaCa-2 cells was carried out using HiPerFect Transfection Reagent (Qiagen, Hilden, Germany) in line with the manufacturer’s standard protocol. In short, cells were seeded in 6-well plates at 4 × 10^5^ cells per well and incubated with HiPerFect Transfection Reagent, Opti-MEM Reduced Serum Medium (Thermo Fisher Scientific, Waltham, MA, USA), and miScript miR-192-5p mimic (Qiagen) for 48 h. As a negative control, MIA PaCa-2 cells were transfected with AllStars NC (Qiagen) instead. Mock transfection was carried out by incubation of cells solely with HiPerFect Transfection Reagent and Opti-MEM Reduced Serum Medium.

### 4.2. Western Blotting

Western blot analysis was used for detection of cellular and exosomal proteins. Cells were dissociated in radioimmunoprecipitation assay (RIPA) buffer with Protease Inhibitor Cocktail (Cell Signaling Technology, Danvers, MA, USA) and homogenized using a Tissue Lyser LT bead mill (Qiagen). Cell lysates were cleared at 14,000× *g* and 4 °C for 45 min. Exosomes were lysed solely in RIPA buffer. For protein quantification, Pierce^TM^ BCA Protein Kit (Thermo Fisher Scientific) was used according to the manufacturer’s standard protocol. Proteins were separated by sodium dodecyl sulfate (SDS) polyacrylamide gel electrophoresis and blotted on a blocked polyvinylidene fluoride membrane (PVDF; Immobilon-P Transfer Membranes; Merck Millipore, Burlington, MA, USA). Primary antibodies were diluted 1:1000 (mouse anti-Alix; 2171; New England Biolabs, Ipswich, MA, USA), 1:400 (rabbit anti-CD63, sc-15363; Santa Cruz Biotechnology, Dallas, TX, USA), 1:5000 (mouse anti-GAPDH, BioLegend 919501; BioLegend, San Diego, CA, USA), 1:500 (rabbit anti-ZEB1, HPA027524; Sigma-Aldrich, St. Louis, MO, USA), and 1:1000 (rabbit anti-ZEB2, ab138222; Abcam, Cambridge, UK). Horseradish-peroxidase linked secondary antibodies were diluted 1:14,000 (anti-rabbit, A6154; Sigma-Aldrich) and 1:130,000 (anti-mouse, A9044; Sigma-Aldrich). Primary antibodies were incubated overnight at 4 °C, secondary antibodies were incubated for 1 h at room temperature. Peroxidase activity was detected using Immobilon Western Chemiluminescent HRP Substrate (Merck Millipore).

### 4.3. Patients

All patients included in this study were recruited by the Department of General, Visceral and Transplantation Surgery of the University Hospital Muenster, Germany, between 2007 and 2018. Written consent for preoperative sampling of blood serum, intraoperative collection of tissue as well as the collection of clinicopathological data including follow-up was given by each patient. The study was approved by the Ethics committee of the University Hospital Muenster (reference numbers: 1IXHai/11.8.2011, 2016-074-f-S) and conducted in accordance with the Declaration of Helsinki. Obtained specimens were stored prospectively in a tissue and blood serum bank of the Department of General, Visceral and Transplantation Surgery. To obviate the risk of treatment-related influences on expression patterns of proteins and miRs, patients who received neoadjuvant chemotherapy were excluded from this study. PDAC patients who were enrolled ahead of the release of the 8th edition of the UICC TNM classification of malignant tumors were restaged for the purpose of the study [52]. Diagnosis and staging were verified by an experienced pathologist. The primary end points of this study were overall survival and recurrence-free survival.

### 4.4. Collection of Tissue, Blood Serum, and Serum Exosomes

Tissue samples were acquired surgically by pylorus-preserving pancreaticoduodenectomy, pancreatic left resection, total pancreatectomy, or excisional biopsy. The tissue was processed into FFPE blocks and stored at room temperature. A representative hematoxylin- and eosin-stained section was examined for every tissue sample. Pathohistological grading and marking was carried out by a pathologist for differentiation of tumoral and PT tissue sections. Each tissue sample diagnosed with PDAC included vital tumor tissue. Whole blood samples were taken from each individual prior to the beginning of therapy and centrifuged at 2600× *g* for 10 min within 30–60 min of collection (Megafuge 1.0 R; Heraeus, Hanau, Germany). Serum supernatants were aliquoted, centrifuged at 12,100× *g* for 10 min (Minispin; Eppendorf, Hamburg, Germany) and stored at −80 °C. For isolation of serum exosomes, serum samples were centrifuged for 20 min at 16,000× *g* (Biofuge 28RS; Heraeus). The supernatant was diluted in D-PBS (Dulbecco’s phosphate-buffered saline; Sigma-Aldrich) and centrifuged twice for 3 h at 100,000× *g* and 4 °C (Sorvall^TM^ WX Ultra 80 ultracentrifuge, TH641 swinging bucket rotor; Thermo Fisher Scientific). In-between the two rounds of ultracentrifugation, the supernatant was aspirated and the leftover pellet resuspended in D-PBS. Lastly, the supernatant was again aspired and the exosome pellet resuspended in PBS and stored at −80 °C.

### 4.5. Detection of Serum Exosomes by Scanning Electron Microscopy

For electron-microscopic detection of exosomes, pieces of silicon wafers were cleaned with ethanol and coated with anti-CD63-antibody (sc-15363, Santa Cruz Biotechnology) at 4 µg/mL overnight. After washing with D-PBS, the wavers were incubated with preparations of serum exosomes for 2 h at room temperature. After an additional wash with D-PBS, attached nanovesicles were fixed with 1% glutaraldehyde for 30 min and washed with distilled water twice. Specimens were dehydrated with increasing concentrations of ethanol, dried under vacuum, and sputter-coated with layers of platinum (2 nm) and carbon. Exosomes were imaged with a LEO 1530 VP scanning electron microscope (Carl Zeiss, Oberkochen, Germany) at a voltage of 5 kV.

### 4.6. Isolation and Quantification of RNA

Total RNA from blood serum was extracted using the miRNeasy Serum/Plasma Kit (Qiagen) according to the manufacturer’s instructions. Total RNA from exosome-enriched isolates and cell culture lysates was extracted using the miRNeasy Micro Kit (Qiagen) and miRNeasy Mini Kit (Qiagen), respectively. For purification of cell-free total RNA, *Caenorhabditis elegans*-miR-39 (miRNeasy Serum/Plasma Spike-In Control; Qiagen) was spiked into serum and serum exosome samples in accordance with the manufacturer’s standard protocol. Tumoral and PT tissue samples were collected by punch excision from deparaffinized and macrodissected microtome section of the FFPE tissue. To obtain total RNA, the tissue was processed through a robotic workstation (QIAcube; Qiagen) using the miRNeasy FFPE Kit (Qiagen) in line with standard protocol. RNA eluates were stored at −80 °C.

### 4.7. Quantification of miR-192-5p-Expression by RT-qRT-PCR

For relative quantification of miR-192-5p, SYBR-Green based RT-qRT-PCR was performed using the miScript PCR system (Qiagen). Total RNA eluates were reverse transcribed to complementary DNA (cDNA) using the miScript II RT Kit (Qiagen) with miScript HiFlex buffer (Qiagen; tissue, serum, and cell culture lysates) or miScript HiSpec buffer (Qiagen; serum exosomes). Generated cDNA was engaged in triplicate in a standard protocol RT-qRT-PCR reaction mixture containing components of the miScript SYBR Green PCR Kit (Qiagen) and miScript Primer Assay for miR-192-5p (Qiagen). The reaction was executed using a Bio-Rad CFX384TM RT PCR cycler (Bio-Rad Laboratories; Hercules, CA, USA). For normalization of miR-192-5p expression in tissue and cells, RNU1A, SNORD68, and SNORD96A were selected based on the testing of multiple housekeeping genes in PDAC and their average gene expression stability as previously assessed by geNorm Software (Biogazelle NV, Zwijnaarde, Belgium) [13]. *Caenorhabditis elegans*-miR-39 was used for normalization of circulating miR-192-5p and exosomal miR-192-5p. MiR expression was quantified relative to housekeeping genes and data were analyzed using the 2^−ΔΔCq^ method [53].

### 4.8. Tissue Microarray and Immunohistochemistry

Tissue microarray blocks of 1 mm were cut from FFPE blocks and sliced into 3 µm slides, automatically stained, and evaluated as previously described [13]. In short, the slides were deparaffinized and incubated with primary antibodies E-cadherin, Vimentin (monoclonal, ready-to-use; Ventana Medical Systems, Tucson, AZ, USA), ZEB1, and ZEB2 (polyclonal, 1:400; Sigma-Aldrich). Counterstaining was performed with Hematoxylin II and Blueing Reagent. Next, samples were covered with a series of alcohol (70–99%), Xylol and Cytoseal XYL (Thermo Fisher Scientific) as a cover medium. Quantification of immunohistochemical staining was performed using ImageJ 1.52 (ImageJ Software, National Institutes of Health, Madison, WI, USA). Images of tissue microarrays were taken at 20× magnification with a predefined number of 1,228,800 square pixels. The Color-Threshold function was subsequently used to mark all areas previously stained by immunohistochemistry. The degree of protein expression in tissue microarrays was stated as the mean percentage of square pixels marked by ImageJ for respective images of every protein.

### 4.9. Statistics

Statistical analysis was performed using IBM SPSS Statistic 24 (IBM Corp., Armonk, NY, USA), GraphPad Prism 7 (GraphPad Software, La Jolla, CA, USA), and Microsoft Excel for Windows 10 (Microsoft Corp., Redmont, WA, USA). Mann–Whitney *U* test and Kruskal–Wallis test were used to test for significant differences between two or more study groups, while the degree of linear correlation between two variables was stated as Pearson’s *r*. For comparison of clinicopathological characteristics, Fisher’s exact test was applied. Log-rank test was utilized to detect statistically significant differences in OS and RfS between the subgroups of histopathologic features of curatively resected patients. The diagnostic potential of miR-192-5p was assessed by construction of ROC curves and calculation of the AUC and corresponding 95% confidence interval. The prognostic value of miR-192-5p was assessed by Kaplan–Meier curves and log-rank test. A Cox proportional-hazards model was applied for the conduction of multivariate survival analysis that included variables with statistical significance from the univariate survival analysis. Statistical significance was assumed at *p* ≤ 0.05.

## 5. Conclusions

Thus far, the clinical role of miR-192-5p in PDAC had not been fully defined. In this study we could demonstrate that miR-192-5p acts as a tumor suppressor on EMT through downregulation of ZEB2. Moreover, the quantification of miR-192-5p in PDAC tissue and serum exosomes alone presented with a diagnostic accuracy comparable to that of established tumor marker CA.19-9 and could be a helpful diagnostic tool as part of a biomarker or miR panel in the future. Finally, downregulation of miR-192-5p in tumor tissue shows great potential as an independent prognostic marker in curatively resected PDAC patients.

## Figures and Tables

**Table 1:** Summary of clinicopathologic data of all patients enrolled in this study.

Category	Total	HC ^1^	CA ^2^	CP ^3^	IPMN ^4^	PDAC ^5^ UICC ^6^			*p* ^7^
						II	III	IV	
*n*	168	19	19	38	11	30	28	23	
**Age (years)**									0.105
Median (range)	64 (26–87)	64 (43–87)	66 (42–79)	60 (34–80)	73 (36–79)	63 (42–82)	67 (48–82)	66 (26–80)	
<65	89	11	8	27	3	17	13	10	
≥65	79	8	11	11	8	13	15	13	
**Gender**									**0.034**
Female	77	10	14	15	8	9	11	10	
Male	91	9	5	23	3	21	17	13	
**Body mass index (kg/m^2^)**									0.187
Median (range)	25 (15.9–44.3)	27.6 (23–36)	26.8 (21.6–33.1)	24.3 (18.5–44.3)	22.4 (15.9–26.8)	24.5 (17.1–34.1)	24.8 (19–33)	25 (16.7–44)	
<25	83	7	5	21	8	15	16	11	
≥25	85	12	14	17	3	15	12	12	
**Smoking**									0.315
No	110	12	14	21	9	21	15	18	
Yes	58	7	5	17	2	9	13	5	
**Alcohol**									0.091
No	150	19	18	29	11	27	24	22	
Yes	18	0	1	9	0	3	4	1	
**Pre-surgical diabetes mellitus**									0.539
No	119	16	16	26	8	21	18	14	
Yes	49	3	3	12	3	9	10	9	
**Pre-surgical chronic pancreatitis**									**<0.001**
No	106	19	17	0	7	24	17	22	
Yes	62	0	2	38	4	6	11	1	
**Pre-surgical CA.19-9** ^8^ **(U/mL)**									**<0.001**
Median (range)	26.1 (0.6–17458)	7.7 (6.8–12.8)	8.7 (1.6–43)	15.9 (2.4–375)	19.1 (10–63.6)	87.4 (0.6–3136)	204.3 (2.6–17458)	321.2 (2.4–17041)	
<30	73	19	9	19	7	8	6	5	
≥30	63	0	3	8	1	17	17	17	
**Pre-surgical CEA** ^9^ **(ng/mL)**									**0.013**
Median (range)	1.5 (0.1–26.7)	1 (0.4–1.3)	1 (0.1–4.6)	13 (0.2–6.3)	0.9 (0.2–7.9)	2.1 (0.2–10.8)	2.1 (0.2–10.5)	3 (0.6–26.7)	
<5	105	19	12	21	8	17	15	13	
≥5	15	0	0	1	0	3	6	5	
**Pre-surgical bilirubin (mg/dL)**									**0.001**
Median (range)	0.6 (0.2–24.3)	0.6 (0.2–1.6)	0.6 (0.2–2.7)	0.5 (0.2–1.4)	0.5 (0.3–3.7)	1 (0.2–10.8)	0.9 (0.3–24.3)	0.9 (0.3–5.9)	
<1.2	128	16	15	36	10	19	16	16	
≥1.2	37	1	3	2	1	11	12	7	

Bold values indicate significance (*p* ≤ 0.05, Fisher’s exact test). ^1^ HC, healthy controls; ^2^ CA, benign cystadenoma; ^3^ CP, chronic pancreatitis; ^4^ IPMN, intraductal papillary mucinous neoplasm; ^5^ PDAC, pancreatic ductal adenocarcinoma; ^6^ UICC, Union for International Cancer Control; ^7^ *p*, *p* value; ^8^ CA.19-9, carbohydrate antigen 19-9; ^9^ CEA, carcinoembryonic antigen.

**Table 2:** Univariate survival analysis of curatively resected PDAC patients’ histopathologic features in regard to overall survival and recurrence-free survival.

Category	Overall Survival (OS)				Recurrence-Free Survival (RfS)			
	*n* ^1^	Predicted Median OS (Months)	95% CI ^2^	*p* ^3^	*n*	Predicted Median RfS (Months)	95% CI	*p*
**Age (Years)**				0.236				0.981
<65	23	19	0.0–40.0		23	9	5.9–12.0	
≥65	19	20	9.5–30.5		19	8	4.8–11.2	
**Gender**				0.841				0.198
Female	14	16	2.5–29.5		14	9	3.5–14.5	
Male	28	20	12.8–27.2		28	8	4.8–11.2	
**Body Mass Index (kg/m^2^)**				0.719				0.562
<25	23	20	14.8–25.2		23	9	6.0–12.0	
≥25	19	19	0.2–37.8		19	8	3.9–12.1	
**Smoking**				0.220				0.979
No	25	19	8,7–29.3		25	8	6.9–9.1	
Yes	17	20	1.3–38.7		17	13	7.3–18.7	
**Alcohol**				0.884				0.709
No	38	20	14.7–25.3		38	9	6.6–11.4	
Yes	4	4			4	2.7		
**Pre-surgical Diabetes Mellitus**				0.613				0.734
No	29	20	12.9–27.1		29	9	5.4–12.6	
Yes	13	12	1.8–22.2		13	8	6.3–9.7	
**Pre-Surgical Pancreatitis**				0.723				0.631
No	28	20	1.2–38.8		28	8	6.3–9.7	
Yes	14	20	15.0–25.0		14	10	7.8–12.2	
**Pre-Surgical CA.19-9** ^4^ **(U/mL)**				0.458				0.607
<30	10	12	5.8–18.2		10	8	0.0–16.3	
≥30	22	31	7.3–54.7		22	9	6.3–11.7	
**Pre-Surgical CEA** ^5^ **(ng/mL)**				0.721				0.517
<5	20	23	5.1–40.9		20	16	7.0–25.0	
≥5	6	31	0.0–62.4		6	9	7.0–11.0	
**Pre-Surgical Bilirubin (mg/dL)**				0.728				0.348
<1.2	24	19	7.4–30.6		24	10	5.5–14.5	
≥1.2	18	20	13.0–27.0		18	8	5.3–10.7	
**UICC** ^6^ **Stage**				0.278				0.171
II	30	22	16.4–27.6		30	11	6.0–15.9	
III	12	12	2.4–21.6		12	8	6.4–9.6	
**T Stage**				1.000				0.710
T1	7	22	6.8–37.2		7	8	4.2–11.8	
T2	9	20	10.7–29.3		9	13	9.7–16.3	
T3	26	20	8.3–31.7		26	8	6.8–9.2	
**Nodal Invasion**				0.333				0.328
N0	5	34	17.5–50.5		5	16	9.6–22.4	
N1	25	19	10.6–2.47		25	8	4.2–11.8	
N2	12	12	2.5–21.6		12	8	6.4–9.6	
**Grading**				**0.024**				0.065
G2	21	28	17.4–38.6		21	13	8.6–17.4	
G3	21	12	7.8–16.2		21	8	6.6–9.4	
**Lymphatic Invasion**				0.480				0.056
L0	25	22	13.8–30.2		25	13	8.8–17.2	
L1	17	16	8.7–23.3		17	8	5.3–10.6	
**Perineural Invasion**				0.347				0.171
Pn0	4	23			4	13		
Pn1	33	20	10.5–29.5		33	9	6.9–11.1	
**Vene Invasion**				0.347				0.107
V0	39	19	11.0–27.0		39	9	6.5–11.5	
V1	3				3			
**Resection Margin**				0.657				0.052
R0	30	22	17.6–26.0		30	11	7.4–14.6	
R1	12	13	4.5–21.5		12	7	5.0–9.0	
**Tumor Size (cm)**				0.919				0.502
<3	23	22	16.0–28.0		23	9	5.6–12.4	
≥3	10	20	2.9–37.1		10	10	1.2–18.8	
**Type of Surgery**				0.389				0.678
PPPD ^7^	31	19	12.4–25.6		31	9	5.5–12.5	
Left ^8^	6	11	0.0–22.5		6	7	4.6–9.4	
Total Px ^9^	5	31	0.0–71.4		5	9	0.0–25.1	
**Post-Operative Chemotherapy**				**0.013**				**0.025**
No	2	4			2	2.7		
Yes	40	20	13.5–26.5		40	9	6.0–12.0	

Bold values indicate significance (*p* ≤ 0.05, log-rank test). ^1^ *n*, number of patients; ^2^ CI, confidence interval; ^3^ *p*, *p* value; ^4^ CA.19-9, carbohydrate antigen 19.9; ^5^ CEA, carcinoembryonic antigen; ^6^ UICC, Union for International Cancer Control; ^7^ PPPD, pylorus-preserving pancreaticoduodenectomy; ^8^ Left, pancreatic left resection; ^9^ Total Px, total pancreatectomy.

**Table 3:** Univariate and multivariate survival analysis of curatively resected PDAC patients in regard to overall survival and recurrence-free survival.

Variable	Subset	Univariate Analysis		Multivariate Analysis	
		HR ^1^(95% CI ^2^)	*p* ^3^	HR (95% CI)	*p*
**Overall Survival**					
UICC ^4^ stage	III/II	1.13 (0.37–3.44)	0.833		
Grading	G3/G2	2.97 (1.06–8.30)	**0.038**	5.41 (1.55–18.9)	**0.008**
Nodal invasion	N1-2/N0	1.36 (0.39–4.73)	0.629		
Lymphatic invasion	L1/L0	1.40 (0.55–3.59)	0.483		
Perineural invasion	Pn1/Pn0	1.56 (0.35–6.98)	0.560		
Vene invasion	V1/V0	0.04 (0.00–28.8)	0.338		
Resection margin	R1/R0	0.66 (0.19–2.29)	0.509		
Tumor size (cm)	≥3/<3	1.28 (0.43–3.80)	0.658		
Type of surgery	PPPD ^5^/other	2.06 (0.77–5.51)	0.152		
Adjuvant chemotherapy	Yes/no	0.04 (0.00–0.60)	**0.021**	0.11 (0.00–1.76)	0.118
Tissue miR–192	High/low	0.32 (0.12–0.86)	**0.024**	0.19 (0.06–0.62)	**0.006**
Peritumoral miR-192	High/low	0.58 (0.19–1.75)	0.333		
**Recurrence-Free Survival**					
UICC stage	III/II	1.33 (0.44–4.03)	0.619		
Grading	G3/G2	1.54 (0.65–3.61)	0.325		
Nodal invasion	N1-2/N0	1.47 (0.49–4.39)	0.492		
Lymphatic invasion	L1/L0	2.15 (0.90–5.12)	0.085		
Perineural invasion	Pn1/Pn0	1.77 (0.40–7.84)	0.455		
Vene invasion	V1/V0	0.36 (0.05–2.77)	0.329		
Resection margin	R1/R0	0.99 (0.38–2.57)	0.982		
Tumor size (cm)	≥3/<3	1.53 (0.54–4.33)	0.427		
Type of surgery	PPPD/other	2.61 (0.98–6.97)	0.055		
Adjuvant chemotherapy	Yes/no	0.05 (0.00–0.85)	**0.038**	0.06 (0.00–1.00)	**0.050**
Tissue miR-192	High/low	0.38 (0.15–0.95)	**0.038**	0.39 (0.16–0.98)	**0.045**
Peritumoral miR-192	High/low	0.34 (0.13–1.03)	0.056		

Bold values indicate significance (*p* ≤ 0.05). ^1^ HR, hazard ratio; ^2^ CI, confidence interval; ^3^ *p*, *p* value; ^4^ UICC, Union for International Cancer Control; ^5^ PPPD, pylorus-preserving pancreaticoduodenectomy.

## Supplementary Materials

The following are available online at https://www.mdpi.com/2072-6694/12/6/1693/s1, Figure S1: Expression of E-cadherin, Vimentin, ZEB1, and ZEB2 in benign and malignant pancreatic cell lines, Figure S2: Whole Western blot images and corresponding intensity ratios, Figure S3: Alix, Table S1: Landscape of trials investigating microRNA-192-5p in human malignancies [27,28,30,31,32,33,34,35,36,37,38,39,40,41,42,43,44,45,46,47,48].
