# Correction: Flammang et al. Tumor-Suppressive miR-192-5p Has Prognostic Value in Pancreatic Ductal Adenocarcinoma. *Cancers* 2020, *12*, 1693

**DOI:** 10.3390/cancers16162825

**Published:** 2024-08-12

**Authors:** Isabelle Flammang, Moritz Reese, Anda J. Ströse, Zixuan Yang, Johannes A. Eble, Sameer A. Dhayat

**Affiliations:** 1 Department of General, Visceral and Transplantation Surgery, University Hospital Muenster, Albert-Schweitzer-Campus 1 (W1), 48149 Muenster, Germany; isabelle.flammang@ukmuenster.de (I.F.); m_rees04@uni-muenster.de (M.R.); anda.stroese@web.de (A.J.S.); yzxbcchina@gmail.com (Z.Y.); 2 Department of Physiological Chemistry and Pathobiochemistry, University of Muenster, Waldeyerstrasse 15, 48149 Muenster, Germany; johannes.eble@uni-muenster.de

## 1. Missing Funding

In the original publication [1], the research award of the Maria Moeller Foundation (no Grant Number) to Anda J. Ströse was not included.

The authors apologize for any inconvenience caused and state that the scientific conclusions are unaffected. This correction was approved by the Academic Editor. The original publication has also been updated.

## 2. Addition of an Author

Anda J. Ströse was not included as an author in the original publication. The corrected Author Contributions Statement appears here.

Anda J. Ströse was involved in the conceptualization, project administration, investigation, and methodology, in the data curation, formal analysis, visualization, in the writing—original draft preparation of a first withdrawn manuscript, and in the funding acquisition by the Maria Moeller Stiftung, Muenster Germany.

The authors apologize for any inconvenience caused and state that the scientific conclusions are unaffected. This correction was approved by the Academic Editor. The original publication has also been updated.
